# Advancement of an Environmentally Friendly and Innovative Sustainable Rubber Wrap Film with Superior Sealing Properties

**DOI:** 10.3390/polym16111499

**Published:** 2024-05-24

**Authors:** Sunisa Suchat, Siwarote Boonrasri

**Affiliations:** 1 Faculty of Science and Industrial Technology, Prince of Songkla University Surat Thani Campus, Surat Thani 84000, Thailand; 2 Faculty of Engineering and Agro-Industry, Maejo University, Chiang Mai 50290, Thailand

**Keywords:** natural rubber, wrap film, nitrosamines, zinc oxide, sulfur, sealing

## Abstract

Common kitchen wraps like plastic and aluminum foil create significant environmental burdens. Plastic wrap, typically made from non-renewable fossil fuels, often ends up in landfills for centuries, breaking down into harmful microplastics. Aluminum foil, while effective, requires a large amount of energy to produce, and recycling it at home can be impractical due to food residue. A promising new alternative, low-nitrosamine rubber wrap film, aims to reduce waste by offering a reusable option compared to traditional single-use plastic wrap. The film is environmentally friendly, durable, and effective in sealing containers and keeping food fresh or crispy. The raw materials used to make the product were studied, namely fresh and concentrated natural rubber latex. No nitrosamines were found in either the fresh or concentrated latex, which is important as nitrosamines are known to be carcinogenic. The absence of nitrosamines in the raw materials suggests that the universal rubber wrap film is safe for use. In this study, the rubber formulation and properties of rubber used to make rubber wrap film were studied. The content of additives affecting the rubber properties was varied to find the optimum rubber formulation for making rubber wrap films. The rubber formulation with the least amount of chemicals that met the following criteria was selected: tensile strength of at least 15 MPa, elongation at break of at least 600%, and nitrosamine content below 6 ppm. It was found experimentally that the optimum rubber formulation for making a translucent rubber film had 0.7 phr zinc oxide and 1.0 phr sulfur. Performance tests revealed the rubber wrap film’s superior sealing capabilities. Its elasticity allows for a tighter fit on containers, effectively conforming to various shapes and creating an optimal seal compared to plastic wrap and aluminum foil. The results of this study provide valuable information for developing a universal rubber wrap film that is safe with low nitrosamines.

## 1. Introduction

In everyday life, we often use plastic wrap or aluminum foil to shield food against contamination by dust, dirt, or insects [1,2,3,4,5]. However, plastic wrap and aluminum foil are easily torn, not durable, and cannot be reused. Aluminum foil production requires significant energy consumption. Moreover, recycling aluminum foil often requires separation from food residue, which can be impractical for home use. Plastic wrap is typically derived from non-renewable fossil fuels [6]. Additionally, most commercially available plastic wrap is not readily recyclable and can persist in landfills for hundreds of years, contributing to microplastic pollution. This results in a large amount of waste that generates pollution and has a negative impact on the environment [7,8]. This study addresses environmental concerns associated with traditional food wrap by developing a novel, environmentally friendly and sustainable rubber wrap film. Utilizing natural rubber, a renewable resource, this innovative film is designed for reusability, minimizing waste and environmental impact. The concept has driven the need for a 100% natural rubber wrap film that is both eco-friendly and safe for consumers. This could reduce the use of plastic, which takes over 600 years to decompose [9,10], by serving as a replacement for plastic wrap and aluminum foil. Natural rubber latex (NRL) can be made into thin films with excellent elasticity and flexibility [11]. In particular, NRL is a natural plant-based material that is never depleted. NRL serves as a critical material in the biomedical field, with its unique properties enabling diverse products for various applications, including tubing, latex foam [12,13], rubber nipples, surgical gloves, and catheters [11].

This experimental study was conducted to develop a new rubber product, a rubber wrap film. The production process is similar to that of other natural rubber products, but the rubber wrap differs from commonly used rubber products such as gloves and finger cots. The elasticity of rubber is such that the restoring force increases with stretching [14], which in turn should, as a hypothesis, lead to a more effective sealing with rubber wrap than with PE film. 

The rubber wrap does not contact food or skin directly, unlike rubber gloves. Therefore, one criterion set for the rubber wrap was to have a nitrosamine content of at most 6 ppm, which is equivalent to 6 µg/g or 0.0006%, a very small amount. Gloves, condoms, and latex nipples for babies are regulated to have low levels of N-nitrosamines, at 10 ppb or 0.000001% [15]. This is because these products contact food, skin, and the baby’s mouth directly for long periods of time. Sweat on the skin and saliva in the mouth can allow N-nitrosamines to enter the body. N-nitrosamines in rubber can be analyzed using GC-MS [16,17]. Zinc diethyldithiocarbamate (ZDEC) is a widely used accelerator in natural rubber latex technology due to its ability to impart desirable properties to the resulting vulcanizates, such as a high tensile strength, elongation at break, and tear resistance [11,18]. However, ZDEC has also been shown to release nitrosamines, which are potentially carcinogenic compounds [19]. This study therefore investigated the possibility of reducing the ZDEC content from 1 phr to 0.5 phr to minimize nitrosamine release. In general, increasing the sulfur content (S) in natural rubber increases the tensile strength. The optimum sulfur content for natural rubber vulcanizates is typically 2.0–2.5 phr [20,21,22]. This study investigated the use of the minimum sulfur content required to achieve satisfactory properties for container-sealing applications. According to theory, zinc oxide is used in rubber products in amounts greater than 3 phr to activate the vulcanization reaction and improve the tensile strength of the rubber [23,24,25]. However, the use of zinc oxide in excess of 1 phr can make white rubber opaque. Therefore, this study used a zinc oxide content below 1 phr to produce translucent rubber wrap film that allows the materials in the sealed container to be seen. The reduction in the ZnO content is consistent with the current trend of reducing ZnO use in rubber products due to its potential toxicity to aquatic organisms [24,26,27,28]. 

This study investigated the rubber compound formulation and properties of rubber suitable for making rubber wrap films. The effects of the choice of the natural rubber latex type, namely concentrated latex or fresh latex, on the formation of nitrosamines in the rubber wrap were investigated. The fresh latex was preserved with NH_3_ and TZ (T is for thiuram disulfide TMTD and Z is for zinc oxide ZnO). The concentrated latex was preserved with high and low ammonia levels (HA and LA) with stabilizers. The rubber compounds with low nitrosamines were formulated and developed to obtain the desired properties in the rubber wrap films for container sealing, to replace plastic wrap or aluminum foil. The amounts of zinc oxide, sulfur, titanium dioxide, and carbon black were varied to study the effects on the properties of rubber. For example, the zinc oxide (ZnO) content was tested at 0.5, 0.7, and 0.9 phr to study the transparency of the product, and the sulfur (S) content was tested at 0.5, 1, and 1.5 phr to study the strength of the product. The selection of a suitable formulation for rubber wrap is defined by threshold values for the tensile strength (15 MPa minimum) and elongation at break (600% minimum), to ensure the usability of an eco-friendly and health-conscious natural rubber wrap for future commercial applications. 

## 2. Materials and Methods

### 2.1. Materials

The compounding ingredients and their suppliers used in this experiment were as follows: zinc oxide from Chemmin Co., Ltd. (Samuthprakarn, Thailand), sulfur (S) from Siam Chemical Public Co., Ltd. (Bangkok, Thailand), zinc diethyldithiocarbamate (ZDEC) and tetramethyl thiuram disulfide (TMTD) from Reliance Technochem Co., Ltd. (Bangkok, Thailand), Lowinox CPL (antioxidant) from Sunny World Chemicals Co., Ltd. (Bangkok, Thailand), and potassium hydroxide from Union Science Co., Ltd. (Chiang Mai, Thailand). Both concentrated latex preserved with high ammonia (HA) and concentrated latex preserved with low ammonia (LA) were obtained from Lucky Four Co., Ltd. (Bangkok, Thailand). The natural rubber latex concentrate used in this study was tested in accordance with ISO 2004:2017 [29] and was found to be free of harmful residues or heavy metals. Five types of standard nitrosamines were used in this study: N-nitrosodiethylamine (NDEA), N-nitrosopyrrolidine (NPYR), N-nitrosodipropylamine (NDPA), N-nitrosopiperidine (NPIP), and N-nitrosodibutylamine (NDBA). The initial concentration of each standard was 2000 ppm. The standards were purchased from S.M Chemical Supplies Co., Ltd. (Bangkok, Thailand).

### 2.2. The Levels of Nitrosoamines in Fresh Latex and Concentrated Latex

In this study, the test samples consisted of standard nitrosamines and dry rubber films made from fresh latex and concentrated latex. Five types of standard nitrosamines were used as follows: NDEA, NPYR, NDPA, NPIP, and NDBA. The initial concentration of each standard was 2000 ppm. To study the sensitivity of the analysis, the concentration of each standard was then diluted to 100 ppm, 25 ppm, 12 ppm, and 6 ppm. The analysis time was 40 min.

The rubber samples used in the test were (1) fresh latex preserved with NH_3_; (2) fresh latex preserved with NH_3_ and with the preservative TZ (T is for tetramethyl thiuram disulfide TMTD at 0.125% and Z is for zinc oxide at 0.125% total); (3) concentrated latex preserved with high ammonia (HA); and (4) concentrated latex preserved with low ammonia (LA) with the preservative TZ. In the rubber industry, ammonia is used to preserve latex. Concentrated latex of type HA has a minimum of 0.6% ammonia in the latex. Concentrated latex of type LA has a minimum of 0.2% ammonia in the latex with the addition of 0.025% TZ [30]. However, in this study, TZ was used at a concentration of 0.125%, higher than the normal 0.025%. The test samples were prepared by drying either concentrated latex or fresh latex, then cutting the rubber into small pieces according to the method for preparing rubber samples for the analysis of nitrosamines as described in Section 2.5.

### 2.3. Rubber Wrap Film: Preparation of the Rubber Compound

The development of a rubber latex formula for a multipurpose container film for general use involves testing rubber formulations for properties that are most suitable for general use while having a minimum amount of chemicals. So, the amounts of substances that affect the properties of rubber were varied, including the amount of zinc oxide (ZnO) set at 0.5, 0.7, and 0.9 phr, and the amount of sulfur set at 0.5, 1, and 1.5 phr, in the formulations of Table 1.

The preparation of compounded latex involves adding latex to a tank and mixing it at around 30 rpm at room temperature (25 °C). KOH, sulfur, ZDEC, Lowinox CPL, and ZnO are added gradually, with 10 min of stirring after each addition. The compounded latex is stirred for another 3 days at the same speed, then tested for total solids, chloroform, and vulcanization level by immersion in toluene.

To make dry rubber sheets, a square glass mold was filled with compounded latex and allowed to dry for 3 days. Next, it was heated in an oven at 120 °C for 20 min and removed from the mold. Finally, the vulcanized rubber was tested for key properties like swelling, 300% modulus, tensile strength, elongation at break, and heat aging.

### 2.4. The Production of Rubber Wrap Film to Cover Containers

**Figure 1:**
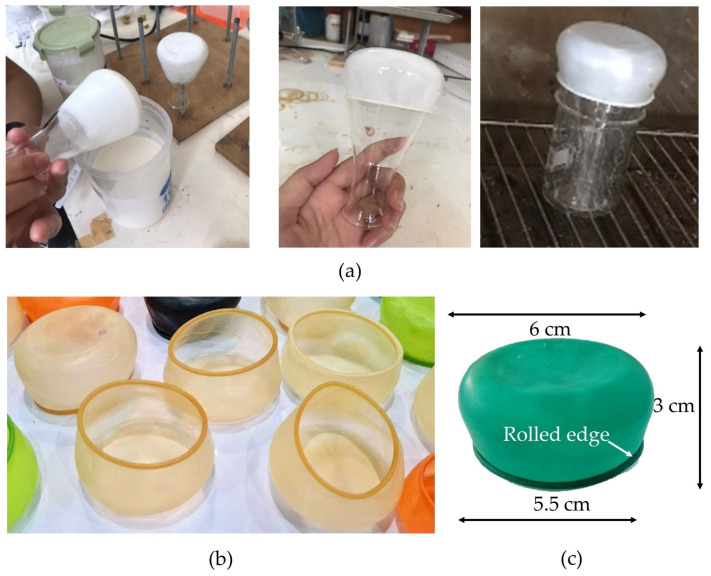
(**a**) The production of rubber wrap film, (**b**) rubber wrap film for covering containers, and (**c**) the size of the rubber wrap film.

The production of rubber wrap film (Figure 1a) used a 125 mL flask by dipping it 2 times for 30 s each time, waiting for the first dip to dry slightly before dipping the second time. Then, the edge was rolled, and the film baked at 120 °C for 20 min. Thereafter, the final product, rubber wrap film, had a truncated conical shape (Figure 1b,c). To test the performance of the rubber wrap in a comparison with PE plastic stretch film and aluminum foil, the color coordinates, water evaporation, and hardness were determined.

The rubber wrap film for covering containers is designed to differ from those for finger cots and gloves. As shown in Figure 1c, the rubber wrap had a truncated conical shape with a top diameter of 6 cm, a bottom diameter of 5.5 cm, and a height of 3 cm. The bottom portion of the workpiece, with a diameter of 5.5 cm, has a rolled edge (bead) for strength to provide a tight seal with the container while the rubber wrap tapers on the sides (Figure 1c). Previous experiments have shown that a cylindrical rubber wrap had a lower sealing capacity than a truncated conical rubber wrap, so the latter type was used in this experiment.

### 2.5. Nitrosamines Testing by Gas Chromatography–Mass Spectrometry (GC-MS)

Gas chromatography–mass spectrometry (GC-MS) is an analytical technique that enables the identification and quantification of organic compounds in various samples. The equipment consists of two main components: a gas chromatograph, which separates the sample into its constituent parts based on their volatility and affinity to the stationary phase, and a mass spectrometer, which measures the mass-to-charge ratios in the fragmentation pattern of each part. The preparation of rubber samples for the analysis of N-nitrosamine levels was based on the conditions of a gas chromatograph–mass spectrometer (GC-MS) as specified in the standards EN 12868:2017, ASTM F1313-90:1999, ISO 29941:2010, and EN 71-12:2013 [31,32,33,34].

For the extraction of rubber samples using the Soxhlet method, rubber articles were cut into pieces of size less than 2 mm × 2 mm. Ten grams of cut pieces was Soxhlet-extracted in dichloromethane at 55 °C, followed by purification with distillation, and dilution to a volume of 2 mL with dichloromethane for analysis.

The condition of the GC-MS was assessed by dissolving the rubber extract in di-chloromethane, a common solvent for organic compounds. Carrier gas at a pressure of 20 psi was used in the analysis by GC-MS for the separation and identification of volatile compounds. The analysis was performed with a Clarus Model 690 gas chromatograph (PerkinElmer, Waltham, MA, USA) coupled to a model SQ8 mass-selective detector. The capillary column used was a PerkinElmer Elite-5MS (5% phenylmethyl polysiloxane with a 30 m × 250 μm ID × 0.25 μm film thickness) (PerkinElmer, Waltham, MA, USA). The injector temperature was 250 °C. The oven temperature was initially held at 60 °C and then increased at 5 °C/min to a final temperature of 250 °C. The carrier gas was purified helium gas. Electron impact (EI) mass spectra were collected at 70 eV ionization voltage over the m/z range 60–600. The electron multiplier voltage was 1350 V. Both ion source and quadrupole temperatures were set at 200 °C. Volatile organic compounds released were identified by computer search in the National Institute of Standard and Technology (NIST) Mass Spectral Library Search Chromatogram.

### 2.6. Rubber Compound Properties

The properties of the rubber compounds were evaluated by three tests: the total solids content, and the degree of vulcanization by immersion in toluene and by the chloroform test. The total solids content (TSC) [11,35] of the rubber compound was determined in strict accordance with ISO 124:2011(E) [36], Rubber latex—Determination of total solids content (ISO, 2011). For the TSC of latex, a sample of 3–5 g of field latex or 2–2.5 g of concentrated latex was accurately weighed using an electric balance with a resolution of 0.0001 g (PR224, OHAUS, Parsippany, NJ, USA) and transferred to an 8 cm diameter Petri dish. The latex was carefully spread evenly across the dish to ensure uniform drying. The sample was then placed in a hot-air oven (UN55, Memmert, Eagle, WI, USA) maintained at 70 °C for a duration of 16 h to achieve complete drying. Subsequently, the samples were transferred to a desiccator for a period of 30 min to facilitate cooling to ambient temperature. The cooled samples were then reweighed using the same balance, and the TSC was calculated using the following Formula (1):

TSC, (%) = [(W2 − W0)/(W1 − W0)] × 100(1)

where W0 is the mass of the empty Petri dish, W1 is the mass of the Petri dish with the latex sample before drying, and W2 is the mass of the Petri dish with the latex sample after drying.

The degree of swelling of latex compounds (SoLC) was determined by immersion in toluene. The test specimen was prepared by dipping a piece of paper into the rubber compound and allowing it to dry for 30 min. The dried film was then cut to a diameter of 20 mm (d0). The film was then immersed in toluene for 30 s with the cap closed. The diameter of the film (d1) was measured after immersion. The degree of cure was then calculated using the following Equation (2):

SoLC, (%) = [(d1 − d0)/d0] × 100(2)

where d0 is the diameter of the film before immersion in toluene, and d1 is the diameter of the film after immersion in toluene.

The degree of vulcanization of the latex compounds (LCs) was assessed using the chloroform number (CN) test. This test involves the coagulation of the LC by mixing it with an equal volume of chloroform. The resulting coagulum is then examined and graded based on its texture, providing a qualitative indication of the extent of vulcanization. The following CN grading system was employed: (1) Unvulcanized: the coagulum is soft, sticky, and easily deformable, indicating the absence of significant cross-linking between rubber molecules. (2) Lightly vulcanized: the coagulum exhibits slight resistance to deformation, suggesting a low degree of cross-linking. (3) Moderately vulcanized: the coagulum displays moderate resistance to deformation, implying a moderate degree of cross-linking. (4) Fully vulcanized: the coagulum is firm, resilient, and resists deformation, signifying a high degree of cross-linking and a well-vulcanized rubber network. The CN test provides a rapid and convenient method for qualitatively evaluating the degree of vulcanization in LCs [11].

### 2.7. Mechanical Properties of Rubber Wrap Film

The degree of swelling in the vulcanized specimens was assessed through a toluene immersion test. Accurately measured specimens (3 cm × 1 cm × 2 mm) were prepared, and their initial weights determined using an analytical balance. These specimens were subsequently immersed in toluene at room temperature. At regular intervals, the specimens were removed, carefully dried, and reweighed until a constant weight was attained. The degree of swelling was then calculated using the following Equation (3):

Swelling, (%) = [(W2 − W1)/W1] × 100(3)

where W1 is the initial weight of the specimen and W2 is the weight of the swollen specimen. 

This test provides a quantitative measure of the extent of cross-linking in the rubber network. A higher degree of swelling indicates a lower degree of cross-linking, as the polymer chains can more readily absorb and expand with the solvent [37]. 

The tensile properties of the rubber wrap films were evaluated in strict accordance with ISO 37:2017 [38]. Dumbbell-shaped test specimens were precisely cut from the films and subjected to tensile testing using a computerized tensile testing machine (Instron 5566, Norwood, MA, USA) equipped with a 1 kN load cell. The crosshead speed of the Instron machine was set to 500 mm/min to ensure consistent testing conditions. From the acquired tensile data, the modulus at 300% elongation (M300) and elongation at break (EB) were meticulously calculated. To ensure statistical validity, the reported values for the tensile properties represent the mean of five independent measurements. To investigate the impact of thermal aging on the mechanical properties, a subset of specimens were subjected to an accelerated aging process within a hot-air oven maintained at 100 °C for a duration of 22 h. Following this aging, the specimens were allowed to cool to ambient temperature for a minimum of 16 h prior to tensile testing, to eliminate any potential thermal effects [11,37].

### 2.8. Performance Properties of Rubber Wrap Film

The performance properties of the rubber wrap film were evaluated by three tests: color measurement (colorimeter), hardness measurement (hardness load), and water evaporation test.

The color of the rubber wrap film was evaluated using a colorimeter (ColorFlex EZ Spec-trophotometer, HunterLab, Reston, VA, USA). At least three test specimens were prepared, each of which was thin enough to be measured by the colorimeter. The L value was measured for each specimen, and the average was calculated. The L value is a measure of lightness, with a value of 0 indicating black and a value of 100 indicating white. In addition, visual observations were conducted by cutting a rubber wrap film into a triangular shape of approximately 2 cm in size. The film was then placed on a white sheet of paper with black letters, and a photograph was taken with a digital camera. The clarity of the black letters was observed.

The hardness of DOZO (rice crackers) was evaluated using a texture analyzer (CTX Texture Analyzer, Brookfield AMETEX, Middleboro, MA, USA). At least three DOZO were placed in a clear plastic cup with a capacity of 18 ounces and a mouth width of 9 cm. A rubber wrap film with a bottom diameter of 5.5 cm, a plastic film, and an aluminum foil were used to cover the cup. The samples were then kept at room temperature for 7 days. The hardness was measured by pressing the center of the sample with a sphere probe at a speed of 1 mm/s. The average hardness was calculated for each sample.

A water remaining content (WRC) test was conducted to compare the efficiency of the rubber wrap film, plastic film, and aluminum foil in preventing water loss. Three Petri dishes with a diameter of 9 cm were prepared, each containing 10 g of water. The dishes were covered with a different type of film, and the weight of the water was measured every day for 7 days. The percentage of the WRC was calculated using the following Equation (4):

WRC, (%) = [(Wt − Wi)/Wt] × 100(4)

where Wt is the final weight of the water and Wi is the initial weight of the water.

### 2.9. Statistical Analyses

Statistical analyses were performed using JMP software v.5.1.2. (SAS Institute, Torrance, CA, USA). The significance level α was set at 0.05. The Tukey–Kramer HSD (honestly significant difference) test was used to compare means and evaluate the significance of differences between treatments. Standard deviation (SD) bars and SD values are presented in figures and tables, respectively.

## 3. Results and Discussion

### 3.1. The Levels of Nitrosoamines in Fresh Latex and Concentrated Latex

**Figure 2:**
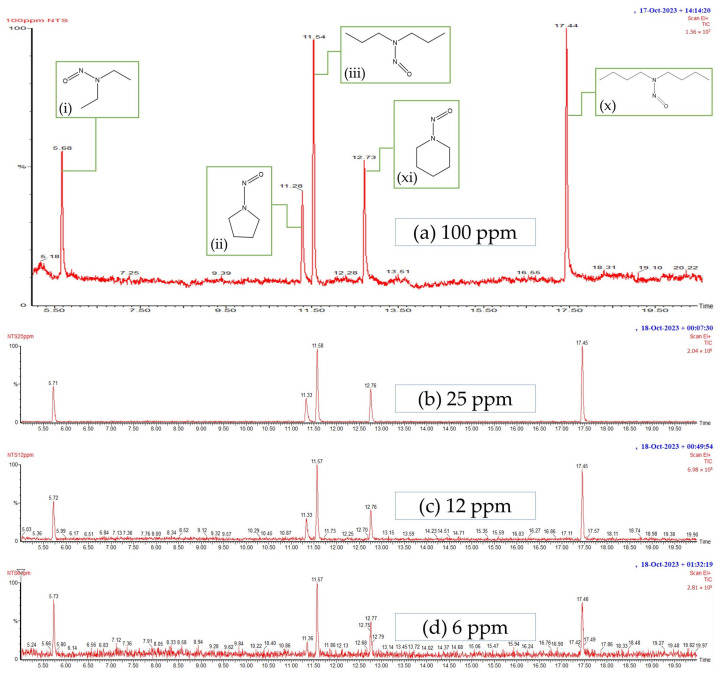
The chromatograms of diluted standard nitrosamines with concentrations of (**a**) 100 ppm, (**b**) 25 ppm, (**c**) 12 ppm (with an extended y-axis scale), and (**d**) 6 ppm (with adjusted y-axis scales for similar maximum peak heights).

The results of a study on the levels of nitrosamines based on five standard nitrosoamines (N-Nitrosodiethylamine (NDEA), N-Nitrosopyrrolidine (NPYR), N-Nitrosodipropylamine (NDPA), N-Nitrosopiperidine (NPIP), N-Nitrosodibutylamine (NDBA)) using a GC-MS showed that all five compounds produced a GC peak at a concentration of 100 ppm, as shown in Figure 2a. Figure 2a shows that the GC peak at position 5.68 (i) belongs to NDEA with a probability of 70.7%, the GC peak at position 11.29 (ii) belongs to N-NPYR with a probability of 80.2%, the GC peak at position 11.54 (iii) belongs to NDPA with a probability of 97.7%, the GC peak at position 12.73 (xi) belongs to NPIP with a probability of 85.56%, and the GC peak at position 17.45 (x) belongs to NDBA with a probability of 92.5%.

When the standard nitrosamines were diluted from a concentration of 100 ppm to 25 ppm, 12 ppm, and 6 ppm, the GC peaks appeared as shown in Figure 2b–d, respectively. As the concentration of the standard nitrosamines decreased, the height of the peak also decreased. At 12 ppm and 6 ppm, the chromatograms were expanded to show the peaks more clearly. Since the peak positions at 12 ppm and 6 ppm match those of the standard nitrosamines at 25 ppm, this indicates that the GC-MS instrument can measure the amount of nitrosamines at a concentration of 6 ppm. An amount of 6 ppm is equivalent to 6 µg/g, which is equal to 0.0006%, a very small amount. Nitrosamines other than the five standard types can be analyzed using a GC-MS instrument if nitrosamines are present in the sample tested. In addition, the researchers believe that if the ZDEC accelerator breaks down into nitrosamines, it will form NDEA. 

**Figure 3:**
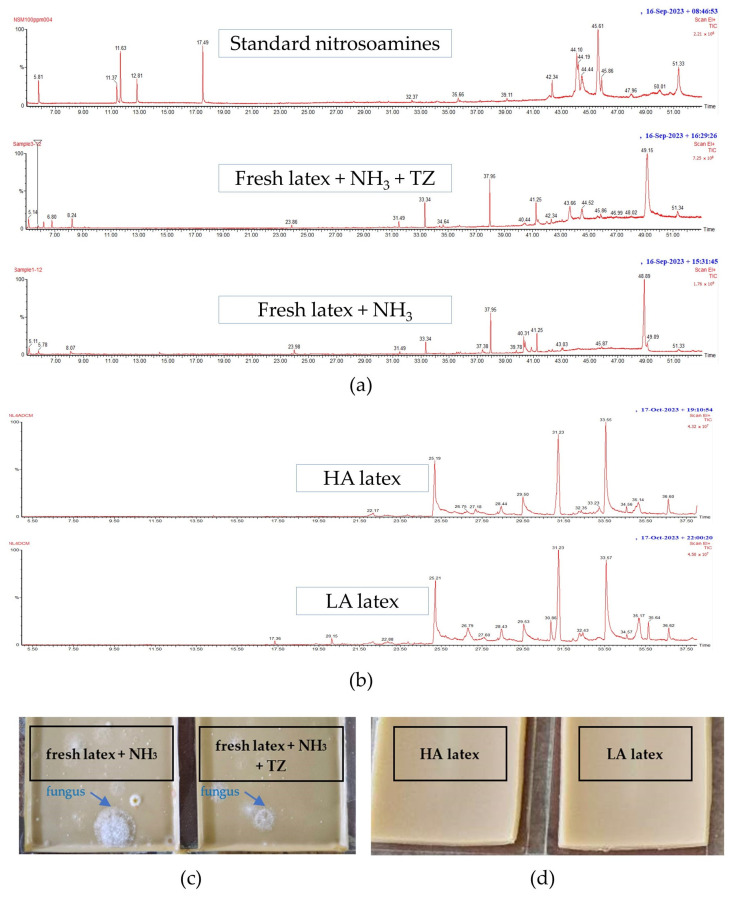
The GC-MS chromatograms of (**a**) fresh latex, (**b**) concentrated latex, (**c**) dried rubber sheets prepared from fresh latex, exhibiting fungal growth, and (**d**) dried rubber sheets prepared from concentrated latex, with no observable fungal growth.

The results of a study on the types of latex used as raw materials in the production of multipurpose wrap films are shown in Figure 3a,b. The four types of latex studied were as follows: (1) fresh latex + NH_3_, (2) fresh latex + NH_3_ + TZ, and the concentrated latexes (3) HA and (4) LA. Figure 3a,b show that neither fresh nor concentrated latex had N-nitrosamines. However, in the next experiment, HA latex was used to make a rubber wrap film. This is because fresh latex may easily undergo fungal contamination in high humidity, as shown in Figure 3c. This is because fresh latex contains starch, sugar, and protein from the latex. In contrast, concentrated latex sheets (HA and LA) do not show fungal growth as these compounds have been removed during the concentration process. This finding is consistent with previous research that has shown that fungal growth is easily facilitated on raw rubber sheets [39]. Fungal growth on rubber sheets can lead to inferior properties and an unpleasant odor from raw natural rubber (NR) and from products made from it, and it can also cause environmental concerns.

Additionally, Figure 3a,b show that a large number of peaks are observed after 20 min. The analysis yielded no detectable N-nitrosamines. It is noteworthy that both the fresh latex and concentrated latex, utilized as raw materials for rubber product manufacturing, exhibited an absence of N-nitrosamines. However, the presence of N-nitrosamines has been confirmed in various finished rubber products, including gloves, balloons, condoms, rubber teats, and soothers [16,17,40].

### 3.2. The Effect of Zinc Oxide (ZnO) Content on the Properties of Rubber Compounds and Mechanical Properties

The degree of swelling of the latex compounds by toluene immersion showed SoLC values of 201 ± 1.5%, 196 ± 1.8%, and 193 ± 1.4% for zinc oxide contents of 0.5, 0.7, and 0.9, respectively. As the zinc oxide content increased, the swelling decreased, indicating a higher degree of vulcanization. The SoLC test is more sensitive than the chloroform test because the chloroform test yielded a grade of 2 for all three formulations. However, the SoLC test can distinguish between the degrees of vulcanization of the different formulations. The TSC test showed values of 50.62 ± 0.31%, 50.52 ± 0.76%, and 50.72 ± 0.53% for zinc oxide contents of 0.5, 0.7, and 0.9, respectively. It is clear that the TSC values in all three formulations are similar.

**Figure 4:**
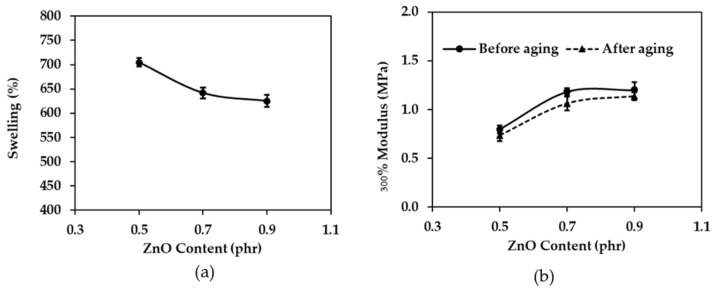
The effect of zinc oxide content on (**a**) swelling and (**b**) 300% modulus before aging and after aging.

In general, swelling is inversely proportional to cross-linking, that is, the more swelling, the less cross-linking. Figure 4a shows that the swelling decreases as the zinc oxide content increases to 0.5, 0.7, and 0.9, phr. This is because the amount of cross-linking between molecules is low at 0.5 phr, allowing toluene to penetrate the rubber chain more than at 0.7 and 0.9 phr. As a result, the swelling percentage with 0.5 phr zinc oxide is higher than that with 0.7 and 0.9 phr zinc oxide. According to Kim, et al. (2015) [41], zinc oxide activates the catalyst, which in turn accelerates sulfur to cross-link with rubber molecules. Therefore, as the zinc oxide content increases, the cross-linking increases and the swelling decreases [42].

**Figure 5:**
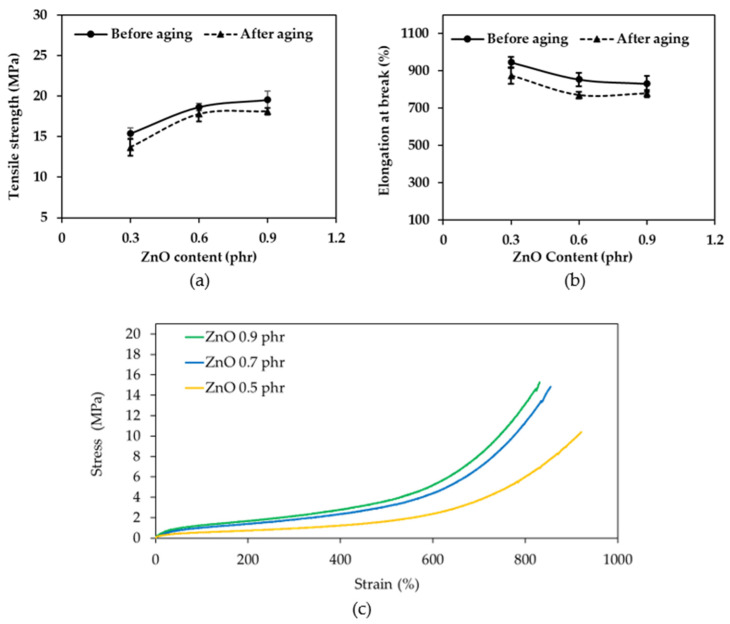
The effect of zinc oxide content on (**a**) tensile strength, (**b**) elongation at break before aging and after aging, and (**c**) stress–strain curves before aging.

Figure 4b shows that the 300% modulus of before aging rubber increases slightly as the zinc oxide content increases. This is because zinc oxide is an activator that accelerates the vulcanization rate of rubber [43,44]. Zinc oxide (ZnO) plays a pivotal role in the vulcanization of rubber by serving as an activator for the cross-linking reactions between sulfur (S) and rubber molecules. This significantly enhances the mechanical properties and durability of rubber products. ZnO exerts its influence by promoting the activity of a key component known as the accelerator, typically denoted by ZDEC. The free radicals resulting from ZDEC cleavage readily engage with sulfur atoms, giving rise to the formation of a transient ZnO-S-ZDEC complex (Figure 8b) [25,44]. This complex acts as a bridge, facilitating the transfer of sulfur atoms from the complex to the rubber molecules. Consequently, covalent bonds form between sulfur and the rubber backbone, leading to the cross-linking of individual rubber chains. The increased cross-linking leads to a higher modulus, as explained previously. The increase in the modulus is accompanied by a decrease in the elongation at break, as shown in Figure 5b. After aging rubber also shows a decrease in the modulus, as heat from the accelerator further accelerates the degradation reaction, resulting in more degradation and a lower modulus.

Figure 5a shows that the tensile strength increases as the zinc oxide content increases for unaged rubber. This is because zinc oxide is a catalyst that increases the efficiency of the vulcanization reaction, as previously mentioned. The increased efficiency of the vulcanization reaction results in more cross-linking between the rubber molecules, which in turn increases the tensile strength. The tensile strength and 300% modulus of rubber with a zinc oxide content of 0.9 phr showed a slight increase from 0.7 phr. This is because the sulfur content was only 0.5 phr, which limited the increase in the tensile strength at a zinc oxide content of 0.9 phr. Based on the results of the initial experiments, ZnO at a content of 0.7 phr was selected for further experiments to investigate the effect of the sulfur content. This was because the initial experiments showed that this content of ZnO resulted in a tensile strength of approximately 14.84 MPa, which is a desirable value. Additionally, this dosage of ZnO is not excessive, which is important for the overall properties of the rubber.

Unaged rubber shows a slight decrease in the elongation at break with the increasing zinc oxide content. This is consistent with the increase in the 300% modulus, which leads to a decrease in the elongation at break. Aged rubber has a lower elongation at break than before aging.

The stress–strain curves (Figure 5c) suggest that the rubber with a higher ZnO content (0.9 phr) has the greatest tensile strength and Young’s modulus, while the 0.5 phr ZnO rubber appears to have the lowest. The elongation at break seems to be highest for the 0.7 phr ZnO rubber, followed by 0.9 phr and then 0.5 phr.

### 3.3. The Effect of Sulfur Content on the Properties of Rubber Compounds and Mechanical Properties

Regarding the properties of the rubber compounds, the SoLC by immersing in toluene gave 195 ± 2.1%, 185 ± 1.2%, and 180 ± 1.9% at sulfur levels of 0.5, 1, and 1.5 phr, respectively. It can be seen that when the amount of sulfur increased, the swelling decreased, indicating a higher level of vulcanization. The test of the vulcanization level by immersing in toluene is more accurate than the chloroform test because when testing with chloroform, it was found that all three formulas obtained grade 2, but when testing the SoLC of the rubber, it could separate the vulcanization level of each rubber formula as mentioned above. The %TSC test showed values of 50.16 ± 0.81%, 51.07 ± 0.28%, and 50.92 ± 0.52% for sulfur contents of 0.5, 1, and 1.5 phr, respectively. It can be seen that the %TSC values for the three formulations of latex are very close. 

**Figure 6:**
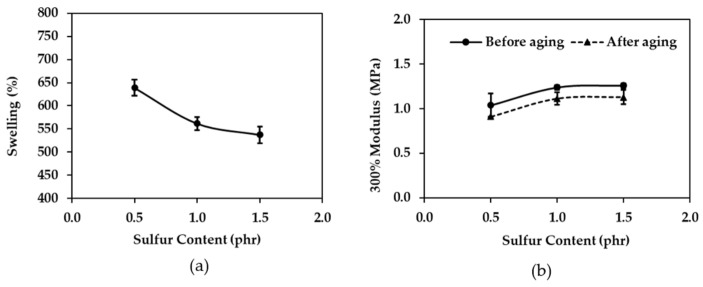
The effect of sulfur content on (**a**) swelling and (**b**) 300% modulus before aging and after aging.

Regarding the mechanical properties of the rubber wrap films, Figure 6a shows that the swelling decreased with the sulfur content. This is because a lower sulfur content results in fewer cross-links. When a specimen is immersed in toluene, the toluene can penetrate the rubber chains more easily. This is consistent with Figure 5, which shows that zinc oxide activates the accelerator, which in turn activates the sulfur. As a result, more cross-links form when the sulfur content increases. Sulfur is a vulcanizing agent that forms cross-links between rubber molecules at reactive sites. When sulfur is added to rubber and heated, vulcanization occurs. As a result, the rubber becomes less soluble in solvents. In general, the higher the sulfur content, the more cross-links form [45].

Figure 6b shows that the 300% modulus increases with the sulfur content for unaged rubber due to the greater cross-link density, as previously mentioned [45]. Rubber that has been aged has a lower 300% modulus than unaged rubber. This is because of the reversion reaction that causes the rubber to deteriorate.

**Figure 7:**
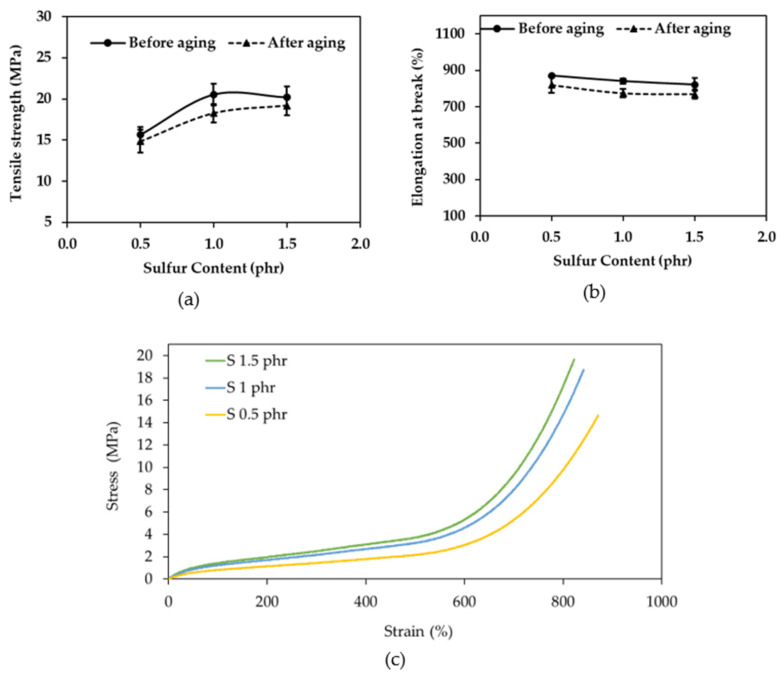
The effect of sulfur content on (**a**) tensile strength, (**b**) elongation at break before aging and after aging, and (**c**) stress–strain curves before aging.

Figure 7a shows that the tensile strength increases with the sulfur content for unaged rubber. The increase in the sulfur content leads to an increase in the cross-link density, which in turn leads to an increase in the tensile strength and modulus, as previously mentioned [45]. This is consistent with the swelling results in Figure 6a, where the higher swelling matches the lower cross-link density and lower tensile strength. Additionally, the tensile strength and 300% modulus of rubber containing 1.5 phr of sulfur exhibited a slight increase compared to the values obtained with 1.0 phr of sulfur. This limited increase is attributed to the low ZnO content of 0.7 phr, which restricts the extent of cross-linking achievable at a sulfur content of 1.5 phr.

Figure 7b shows that the elongation at break decreased with the sulfur content for unaged rubber. This is the opposite of the trend for the 300% modulus, as shown in Figure 6b. This is because the increasing cross-link density results in stiffer rubber, which reduces the elongation at break. Aged rubber, on the other hand, is softer and more ductile after being heated during the accelerated aging process. As a result, the tensile strength of aged rubber is lower than that of unaged rubber. However, all three formulations of rubber, both aged and unaged, meet the requirements for rubber wrap films.

As the sulfur content increases from 0.5 phr to 1.5 phr in the rubber, the stress–strain curves (Figure 7c) should generally show an increase in the tensile strength and Young’s modulus. This is because more sulfur leads to more cross-links between rubber chains. These cross-links restrict the mobility of the chains under stress, requiring a greater force to deform the rubber, resulting in a higher tensile strength. A stiffer material with a higher Young’s modulus would also be expected due to the restricted chain movement.

**Figure 8:**
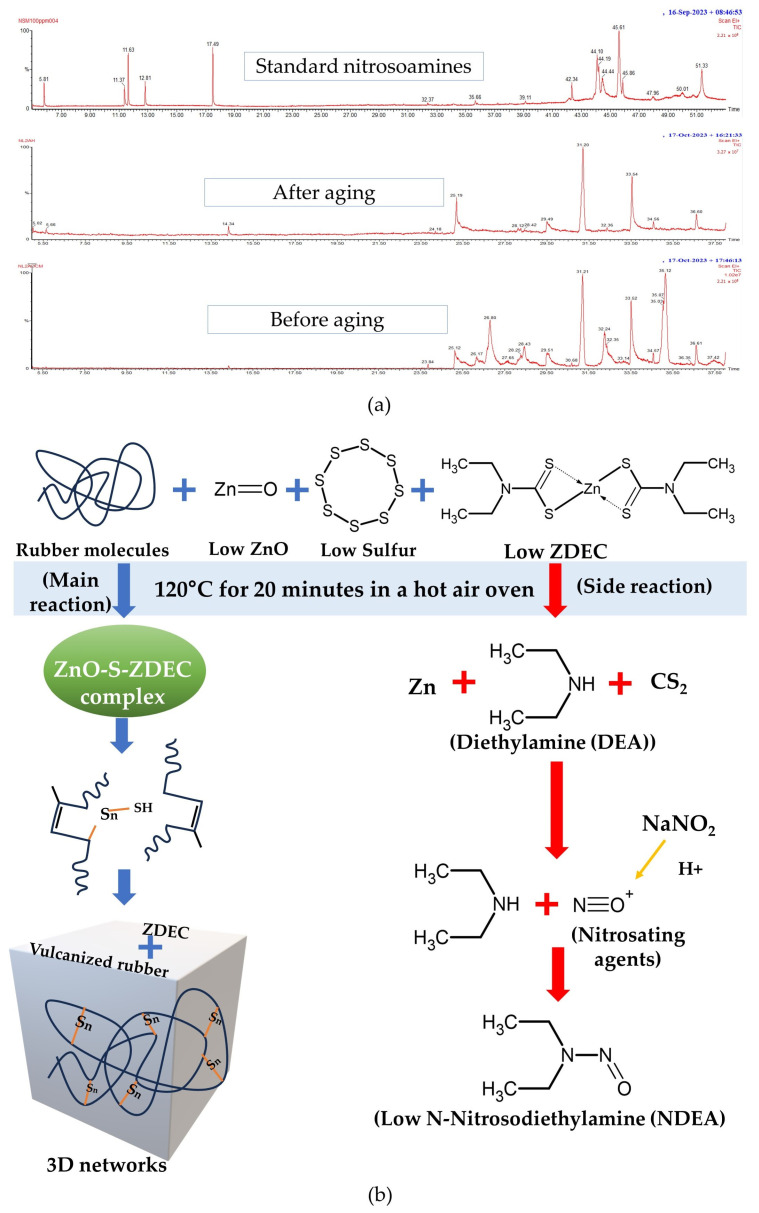
(**a**) The levels of N-nitrosamines in a formulation that used a low-sulfur vulcanization system, before aging and after aging, and (**b**) the proposed mechanism of nitrosamine formation in rubber.

A formulation with 1.0 phr of sulfur and 0.7 phr of ZnO was selected for testing to determine the levels of N-nitrosamines both before and after aging. The results are shown in Figure 8.

Figure 8a shows that no N-nitrosamines were detected in the formulation with 1.0 phr of sulfur and 0.7 phr of ZnO, either before or after aging. After 20 min, peaks were observed, but the analysis showed that they were not N-nitrosamines. Based on the results, a formulation with 1.0 phr of sulfur was selected for further testing because it had the lowest chemical content and met the requirements.

Current research has not shown a link between the use of natural rubber gloves and cancer in humans. There is also no clear evidence of how N-nitrosamines are formed in rubber from ZDEC accelerators. However, it is thought that they can be formed from the use of accelerators (that contain secondary amines in their structure) in the rubber compound used to make gloves. Types of accelerators that may cause N-nitrosamines include ZDMC: zinc dimethyldithiocarbamate, OTOS: N-oxydiethylene -thiocarbamyl-N-oxydiethylsulfenamide, DTDM: 4,4′-dithiodimorpholine, and TMTD: tetramethyl thiuram disulfide. N-nitrosamines are a type of N-nitroso compound that are formed from the reaction of secondary amines with nitrogen oxides [46]. Secondary amines may be formed from the breakdown of accelerators during the vulcanization of rubber at temperatures of approximately 100–150 °C. The breakdown may occur at a level of 0.0000010%, which is a very small amount. Previously, many researchers thought that ZDEC breaks down into secondary amines, but there is no clear research showing how N-nitrosamines are formed in rubber compounds from ZDEC accelerators. Shi et al. (2021) proposed a mechanism for the ZDMC (zinc dithiocarbamate) accelerator during rubber vulcanization [44]. However, this mechanism does not show that ZDMC breaks down into secondary amines (Figure 8b Main reaction). In addition to the fact that the formation of secondary amines in rubber compounds is relatively difficult, the formation of nitrogen oxides is also difficult in rubber compounds. Nitrosating agents may be derived from NaNO_2_, which is not typically added to rubber products. Additionally, NaNO_2_ must be in an acidic environment to react and become a nitrosating agent (Figure 8b Side reaction) [47]. However, TMTD and ZDEC are still used as accelerators in latex products and in the research of new rubber products [48,49,50,51]. Moreover, zinc diethyldithiocarbamate, a disulfiram metabolite, exhibits robust anti-cancer activity in vitro, potentially through mechanisms linked to its role in disulfiram’s anti-alcoholism action [52].

As shown in Figure 8b, the main reaction of the vulcanization process is the formation of a complex between zinc oxide (ZnO), sulfur (S), and zinc diethyldithiocarbamate (ZDEC). This reaction occurs when the rubber is heated to 120 °C for 20 min in a hot-air oven. The sulfur atoms in the ZnO-S-ZDEC complex cross-link the rubber molecules together [25,44], forming a three-dimensional network structure. After vulcanization is complete, the ZDEC is released back to its original form.

Regarding the Side reaction, the proposed mechanism for the formation of nitrosamines in rubber involves the reaction of secondary amines with nitrosating agents (NO). However, the direct reversal of zinc diethyldithiocarbamate (ZDEC) back to its original components—diethylamine (DEA), carbon disulfide (CS₂), and zinc (Zn)—is not a straightforward process under typical conditions. ZDEC is a stable compound, and the energy required to break bonds in the molecule is high. There are several potential approaches to achieve a partial or indirect decomposition of ZDEC, depending on the desired outcome and specific conditions [53,54,55]. These include thermal decomposition on heating ZDEC at a high temperature (around 180 °C or higher), which can lead to its decomposition. However, this approach is likely to yield a complex mixture of products beyond just DEA, CS₂, and Zn. The specific products and their relative amounts will depend on the exact temperature and other factors like the pressure and presence of other chemicals. An alternative is acidic hydrolysis by treating ZDEC with strong acids like hydrochloric acid (HCl). This reaction can potentially lead to the formation of DMA, CS₂, and Zn salts. However, the reaction might not be complete, and other byproducts could be formed depending on the specific acid used and on the reaction conditions. In addition, the formation of nitrosating agents in rubber is a relatively difficult process [56,57,58], as mentioned previously. Nitrosating agents may be contaminants, non-rubber compounds, or they may have formed by oxidation. Further experiments will be conducted later to understand this process better.

### 3.4. Results on the Performance of Rubber Wrap Films in Actual Use

To measure the color of a rubber wrap film, the reflectance of light was measured first. This measurement provides a lightness (L) on a scale from 0 (black) to 100 (white). It is a unitless quantity representing relative darkness or lightness. The thickness of the specimen was measured before the reflectance measurement. The thicknesses of rubber wrap films with zinc oxide contents of 0.5, 0.7, and 0.9 were measured to be 0.36, 0.29, and 0.28 mm, respectively.

**Figure 9:**
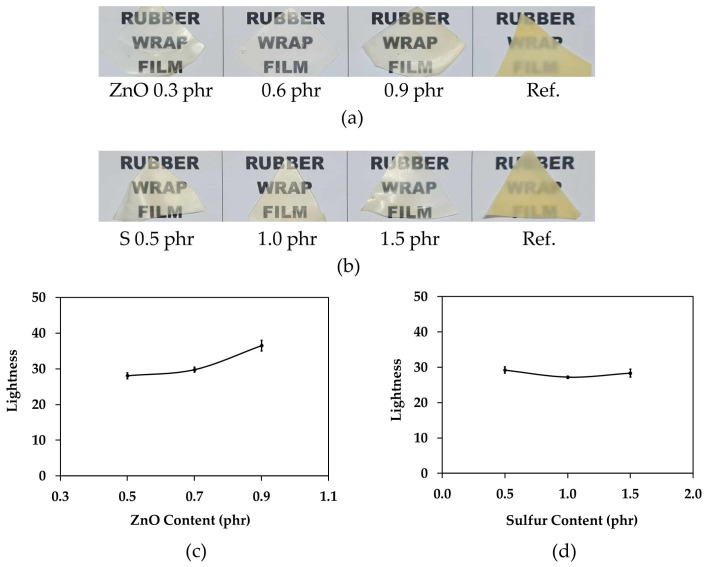
Effect of (**a**) zinc oxide content and (**b**) sulfur content on visual observation; and effect of (**c**) zinc oxide content and (**d**) sulfur content on lightness color coordinate of rubber wrap films.

Visual observation (Figure 9a) showed no difference in the translucence by zinc oxide content. However, Figure 9c shows that the lightness increased at a zinc oxide content of 0.9 phr. This suggests that ZnO increased the reflectance. In Figure 9b, visual observation showed that there was no difference in the transparency between rubber wrap films by sulfur content. The color measurements (Figure 9d) also showed that increasing the sulfur content did not affect the lightness because of the reflectance contribution of sulfur is lower than that of zinc oxide. The experiment is consistent with theory [23]. It is known that a ZnO content greater than 1 phr will make white rubber opaque. This can be observed from the reference sample (ref.), which is a general-purpose glove. The ref. sample may contain more than 1 phr of ZnO, making it difficult to see the letters below the rubber film. This is in contrast to rubber wrap films with less than 1 phr ZnO, which are more translucent. Also, reducing the use of ZnO from the normal content of 3 phr to 0.7 phr can reduce toxicity to aquatic organisms, as previously mentioned. The yellow color of rubber films is due to the presence of carotenoids in natural rubber latex [59].

**Figure 10:**
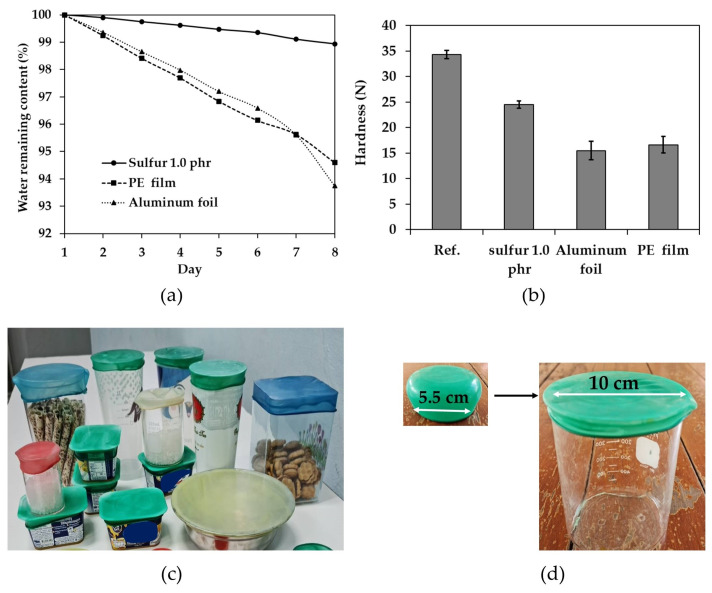
The results of (**a**) the water remaining content and (**b**) the hardness of stored cracker, when comparing rubber wrap film, PE plastic film, and aluminum foil. (**c**) The rubber wrap film can be used to seal any shape of container, and (**d**) rubber wrap film before and when used to cover container.

Figure 10a shows the results of a water evaporation test. In the test, 10 g of water was placed in a Petri dish and sealed with a rubber wrap film containing 1.0 phr sulfur and ZnO at 0.7 phr. The Petri dish was then weighed daily for one week. The results showed that the rubber wrap film sealed the Petri dish more tightly than the PE plastic film or aluminum foil. This is due to the tapered shape of the rubber wrap film, which has a narrower opening than the Petri dish. It was observed that a rubber wrap film with a base diameter of 5.5 cm was able to seal a Petri dish with a diameter of 9 cm. The calculated stretch ratio was approximately 163%. A rubber with a high elongation will have a high tensile force due to the greater amount of energy stored in the stretched rubber [14].

Figure 10b shows that the initial hardness of the snack was 34.45 (Ref.). When the rice crackers were removed from the package and closed with various materials for 7 days, the hardness decreased significantly. This was attributed to the snack being exposed to air and accumulating moisture. The hardness of the snack stored in a plastic cup and closed with a rubber wrap film containing 1.0 phr sulfur and 0.7 phr ZnO, as compared to the PE plastic film and aluminum foil, was tested using a Brookfield Ameter. The results showed that the snack stored in a plastic cup and closed with a rubber wrap film had the highest hardness and was the crispest. In real-world tests, it was found that the rubber wrap film, which is translucent, can seal the mouth of a container more tightly than PE plastic film or aluminum foil. This is because rubber is elastic, which allows the rubber wrap film to seal the mouth of the container more tightly. The rubber wrap film containing 1.0 phr sulfur and 0.7 phr ZnO had a tensile strength of approximately 18 MPa, which is sufficient to seal the mouth of a container tightly. If the tensile strength is greater than this, it will require more force to stretch the rubber to seal the mouth of the container. In addition, one possible mechanism by which food films preserve food crispness is by regulating the moisture absorption of the food. Excessive moisture can lead to sogginess and loss of crispiness, particularly for starchy foods like rice crackers. Moisture plays a critical role in the texture of starch-rich foods. Excessive moisture exposure leads to the softening of the starch network and a significant loss of crispness. This is consistent with the findings of previous studies (Figure 10a), which showed that the rubber film retained moisture better than plastic wrap and aluminum foil. The performance tests reveal the rubber wrap film’s superior sealing capabilities compared to plastic wrap and aluminum foil, potentially extending food shelf life and preserving crispness. 

In addition, rubber wrap film is easier to use than plastic wrap or aluminum foil as food wrapping. It can be used to seal any shape of container, including rectangular, triangular, circular, etc., as shown in Figure 10c, due to the high elasticity of rubber. The rubber wrap film is designed to be different from gloves and finger cots. The sides of the rubber wrap film are tapered to allow the rubber wrap film to be tightly secured on the container. As shown in Figure 7b, the elongation at break of the rubber wrap film is more than 800%. However, in actual use, the rubber will not be stretched to 800%. As shown in Figure 10d, a rubber wrap film with a bottom diameter of 5.5 cm can seal the opening of a container with a 10 cm diameter: the calculated stretch is approximately 182%. This indicates that a rubber wrap film with a diameter of 5.5 cm can seal the opening of containers with a size of 7–10 cm or a stretch of approximately 127–182%. If the opening of a container with a size less than 7 cm is sealed, the sealing force will be low due to the small elongation of the beaded edge [14]: it is necessary to change to a smaller size rubber wrap film to cap a smaller container, to maintain a tight seal. The rubber wrap can cover the mouth of the container of any shape, whether it is a hexagon, triangle, circle, etc. The rubber wrap film is currently in a patent application, in Thailand. While the current study presents a promising alternative with reusability and potentially lower nitrosamine content, a complete picture of its environmental impacts requires a broader analysis of its life cycle. Further research is needed to assess the environmental impacts of natural rubber latex harvesting, processing, film production, use, and disposal. 

## 4. Conclusions

This study investigated a novel application of natural rubber latex, namely as a reusable wrap film designed for covering containers. The production process for this rubber wrap film is similar to other natural rubber latex products. However, the formulation differs from those used in widely available items like gloves, condoms, and baby nipples. Since the wrap film does not directly contact food or skin, regulations regarding the nitrosamine content are less stringent, allowing up to 6 ppm. Analysis of both fresh and concentrated natural rubber latex as raw materials revealed no contribution to the nitrosamine levels, ensuring the final product remained below the 6 ppm limit. The focus of this study was to develop a rubber formulation specifically tailored for use as a container wrap film. This formulation prioritized minimal chemical content while achieving the essential physical properties. The target criteria included the following: tensile strength of at least 15 MPa, elongation at break of at least 600%, and nitrosamine content below 6 ppm. Evaluation identified a suitable formulation for producing a translucent rubber wrap film. This formulation incorporated 0.7 phr of zinc oxide and 1.0 phr of sulfur, achieving the desired properties with no detectable nitrosamine. Compared to traditional PE plastic wrap and aluminum foil, the newly developed rubber wrap film demonstrated superior sealing capabilities when closing container mouths. Additionally, the rubber film offered greater user convenience. Its adaptability allowed it to effectively cover containers of various shapes, including hexagonal, triangular, and circular. The tapered design, a key distinction from finger cots and gloves, ensures a tight fit around container edges. This study demonstrates the potential for formulating safe, environmentally friendly, and commercially viable natural rubber wrap films for future consumer use.

## Figures and Tables

**Table 1:** The compound formulations of rubber wrap film used in the present study.

Ingredient	Content, phr ^a^				
in Formulation	1	2	3	4	5
60% HA latex	100	100	100	100	100
10%KOH	0.2	0.2	0.2	0.2	0.2
ZnO	0.5	0.7	0.9	0.7	0.7
Lowinox CPL	0.5	0.5	0.5	0.5	0.5
ZDEC	0.5	0.5	0.5	0.5	0.5
Sulfur	0.5	0.5	0.5	1	1.5

^a^ Parts per hundred rubber (phr).

## Data Availability

The original contributions presented in the study are included in the article. Further inquiries about the data can be directed to the corresponding authors.
